# Association between Traffic-Related Black Carbon Exposure and Lung Function among Urban Women

**DOI:** 10.1289/ehp.11223

**Published:** 2008-06-04

**Authors:** Shakira Franco Suglia, Alexandros Gryparis, Joel Schwartz, Rosalind J. Wright

**Affiliations:** 1 Department of Environmental Health, Harvard School of Public Health, Boston, Massachusetts, USA; 2 Department of Hygiene and Epidemiology, University of Athens, Athens, Greece; 3 Channing Laboratory, Brigham and Women’s Hospital, Boston, Massachusetts, USA

**Keywords:** air pollution, lung function, particles, traffic

## Abstract

**Background:**

Although a number of studies have documented the relationship between lung function and traffic-related pollution among children, few have focused on adult lung function or examined community-based populations.

**Objective:**

We examined the relationship between black carbon (BC), a surrogate of traffic-related particles, and lung function among women in the Maternal–Infant Smoking Study of East Boston, an urban cohort in Boston, Massachusetts.

**Methods:**

We estimated local BC levels using a validated spatiotemporal land-use regression model, derived using ambient and indoor monitor data. We examined associations between percent predicted pulmonary function and predicted BC using linear regression, adjusting for sociodemographics (individual and neighborhood levels), smoking status, occupational exposure, type of cooking fuel, and a diagnosis of asthma or chronic bronchitis.

**Results:**

The sample of 272 women 18–42 years of age included 57% who self-identified as Hispanic versus 43% white, and 18% who were current smokers. Mean ± SD predicted annual BC exposure level was 0.62 ± 0.2 μg/m^3^. In adjusted analysis, BC (per interquartile range increase) was associated with a 1.1% decrease [95% confidence interval (CI), −2.5% to 0.3%] in forced expiratory volume in 1 sec, a 0.6% decrease (95% CI, −1.9% to 0.6%) in forced vital capacity, and a 3.0% decrease (95% CI, −5.8% to −0.2%) in forced mid-expiratory flow rate. We noted differential effects by smoking status in that former smokers were most affected by BC exposure, whereas current smokers were not affected.

**Conclusion:**

In this cohort, exposure to traffic-related BC, a component of particulate matter, independently predicted decreased lung function in urban women, when adjusting for tobacco smoke, asthma diagnosis, and socioeconomic status.

It is well documented that air pollution is associated with a number of respiratory and cardiovascular adverse health effects (Beelen et al. 2008; Downs et al. 2007; Katsouyanni et al. 1997; Schwartz 1996; Schwartz et al. 2005). Many of these effects seem more strongly associated with particles from traffic (McCreanor et al. 2007; Schwartz et al. 2005), which are rich in elemental carbon and are the principle source of ultrafine particle exposure. Concentrations of traffic-related pollutants [i.e., particulate matter (PM), black carbon (BC), nitrogen dioxide] have been found to increase respiratory symptoms among children (Kim et al. 2004). Among children, long-term exposure to air pollution (Schwartz 1989, 2004) and indicators of increased traffic exposure (Brunekreef et al. 1997; Gauderman et al. 2007; Wjst et al. 1993) have been associated with reduced levels of lung function, a more objective measure of respiratory health.

A few studies have examined the relationship between lung function and long-term exposure to traffic pollution among adults. Among those that have, reduced lung function has been demonstrated among adults with increased traffic exposure (Evans et al. 1988; Kan et al. 2007; Karita et al. 2001; Schikowski et al. 2005; Sekine et al. 2004), although some studies have found no association (Nakai et al. 1999; Tollerud et al. 1983). Furthermore, these studies have focused largely on occupational populations (Evans et al. 1988; Karita et al. 2001; Raaschou-Nielsen et al. 1995; Tollerud et al. 1983), whereas data on community-based populations remain sparse (Kan et al. 2007; Schikowski et al. 2005; Sekine et al. 2004).

In a community-based study of women 30–59 years of age in Tokyo, Japan, Sekine et al. (2004) reported decreased lung function among women living in districts with high traffic density (> 20,000 vehicles) compared with women living in districts with lower average traffic density. A recent community-based study in four communities in the United States demonstrated decreased lung function among middle-age women related to increased traffic density and closer proximity to major roadways (Kan et al. 2007). We expand this work by examining BC from mobile sources estimated using a validated spatiotemporal land-use regression model as a surrogate of traffic particles. BC from traffic sources has been associated with increased risk of asthma and bronchitis among children (Kim et al. 2004), and elemental carbon has been associated with decreased growth in lung function, also among children (Gauderman et al. 2004).

Our goal in this study was to examine the relationship between long-term exposure to BC from traffic sources, a component of PM, and lung function among women of childbearing age followed in a prospective urban community-based cohort study of mother–child pairs.

## Materials and Methods

### Study population

Women were voluntary participants in the Maternal–Infant Smoking Study of East Boston, a prospective cohort originally designed to study the effects of pre-and postnatal tobacco smoke exposure on childhood lung growth and development and respiratory health. The study has been described in detail previously (Hanrahan et al. 1992). In brief, pregnant women receiving prenatal care (<20th week of gestation) at an urban community health center in Boston, Massachusetts, were recruited during the study’s enrollment period, between March 1986 and October 1992. A total of 848 women delivered a full-term infant and remained eligible for postnatal follow-up. A random sample of women was approached for lung function testing; 272 women completed the test and had their home addresses successfully geocoded. There were no significant differences between those who participated in the lung function assessment and those who did not, based on sociodemographics, asthma diagnosis, or tobacco smoke exposure. All participants gave voluntary written consent at the start of the study. The study was approved by the human studies committees at the Harvard School of Public Health, Brigham and Women’s Hospital, and the Beth Israel Deaconess Medical Center.

We estimated exposure to BC based on residence assessed at the time of enrollment, which occurred between 1986 and 1992. To predict residential BC level, we developed a validated spatiotemporal land-use regression model to predict 24-hr measures of traffic-related pollution exposure using data from more than 80 locations in the greater Boston area. Three-quarters of the monitoring sites were residential; the rest were at commercial or government facilities. The data consist of > 6,021 pollution measurements from 2,127 unique exposure days. A detailed description of all exposure data sources is provided by Gryparis et al. (2007). Predictors in the regression were the BC value at a central stationary monitor (to capture average concentrations in the area on that day), meteorologic conditions and other characteristics (e.g., weekday/weekend) of a particular day, and measures of the amount of traffic activity [e.g., geographic information system (GIS)-based measures of cumulative traffic density within 100 m, population density, distance to nearest major roadway, percent urbanization] at a given location. A cumulative traffic density measure, within a 100-m buffer, is recorded once per location. This was the sum of traffic counts on all road segments within 100 m, multiplied by the length of each road segment. We used spline regression methods to allow these factors to affect exposure levels in a potentially nonlinear way. Finally, we used thin-plate splines, a two-dimensional extension of regression splines, to model longitude and latitude and capture additional spatial variability unaccounted for after including our deterministic spatial predictors in the model. This approach is a form of geoadditive model (Kammann and Wand 2003) for daily concentrations of PM levels. We had complete information on all of these factors for 2,114 of the 2,127 unique exposure days. We fit a separate model for the warm (May–October) and cold (November–April) seasons. The *R*^2^ of the model (over both seasons) was 0.82. The model was separable into components varying over time and components varying over space. For long-term average exposure, we took the prediction of the spatial component plus the long-term average of the temporal predictors. For the purposes of these analyses, we used the average of the two seasons as a measure of average BC exposure. If participants moved within a year from the enrollment date, we calculated an average BC measure for all addresses. This model has been previously used to associate BC at home addresses with mortality risk in adults (Maynard et al. 2007) and with cognitive function in children (Franco Suglia et al. 2007).

We measured lung function using a Morgan spirometer (P.K. Morgan, Andover, MA, USA) during the enrollment period (March 1986–October 1992), before 20 weeks of gestation. We measured standing height without shoes before spirometry. The lung function protocol was developed by an experienced pulmonologist and pulmonary function technologist. A trained pulmonary function technologist instructed the women during testing and monitored the flow-volume curve to ensure good effort. Maneuvers were repeated to obtain three acceptable curves; we stored data from each technically acceptable effort following established guidelines (American Thoracic Society 1995). We measured forced expiratory volume in 1 sec (FEV_1_), forced vital capacity (FVC), and forced mid-expiratory flow rate (FEF_25–75_%) from the best acceptable blow (American Thoracic Society 1995).

We ascertained detailed data on race/ethnicity and socioeconomic status (SES) based on education level through standardized questionnaires administered at baseline and clinic follow-up visits, as previously described (Hanrahan et al. 1992). We asked participants about their smoking status on a standardized questionnaire and classified them as current smokers, former smokers, or nonsmokers. We obtained a urine specimen to determine creatinine-corrected cotinine levels, as previously detailed (Hanrahan et al. 1992). We classified participants as never smokers if they reported on the standardized questionnaire that they had never smoked and if their urine cotinine levels were < 200 ng cotinine/mg creatinine (Hanrahan et al. 1992). If the self-report of nonsmoking was contradicted by the urine cotinine, we classified the participant as a current smoker. We also assessed environmental tobacco exposure, defined as a smoker living in the participant’s home.

We assessed occupational exposure history by asking participants whether they ever worked in a dusty job (yes/no), for how long they held that job, and the severity of the dust exposure (mild, moderate, or severe). We defined occupational dust exposure as dust exposure at work for > 2 years or dust exposure that was reported by participants as being moderate or severe. Participants were also asked about the type of cooking fuel used in their homes (gas or electric) and whether they had lived in their residence for at least 2 years.

We ascertained information on respiratory illness history using the standardized American Thoracic Society Division of Lung Diseases questionnaire (Ferris 1978). We determined history of asthma based on a report of physician-diagnosed asthma. We also asked participants whether they had ever been diagnosed with chronic bronchitis.

And finally, there may be factors operating at the aggregate (i.e., neighborhood or community level) that may be more important than individual-level factors in predicting exposures to environmental hazards including air pollution (O’Neill et al. 2003). We examined neighborhood-level SES markers, including percent unemployed within census tracts and living in a census tract with > 20% of households below the federal poverty guidelines. Notably, although participants resided in 43 different census tracts, 48% of the participants resided in five census tracts.

### Statistical analyses

We report estimated effects on pulmonary function using percentage of predicted lung function (based on height, weight, age, and race/ethnicity) as our independent variable. To obtain the predicted lung function values for each woman, we regressed the log-transformed lung function measures against log height, age, log weight, and race/ethnicity. Because we used BC as a surrogate for traffic-related PM exposure, which includes more than just carbon particles, it did not make sense to report results on a unit mass basis. Instead, we report estimated effects of predicted BC per interquartile range (IQR).

We estimated the effect of predicted BC on lung function by linear regression while adjusting for standard control variables, which included year of assessment, age, race/ethnicity (white vs. other race/ethnicity), and education as a marker of individual-level SES (model 1). To assess the potential for confounding, we examined the sensitivity of those results to further adjustment for smoking status (model 2) and a diagnosis of asthma and/or chronic bronchitis (model 3). Finally, we used multilevel modeling to take into account the neighborhood-level SES markers (Goldstein 2003). We also considered tertiles of BC exposure to assess for a threshold effect or an exposure–response relationship.

We next ran a series of stratified regressions as further sensitivity analyses. We first stratified our analyses by length of residence. Second, we conducted separate regression models for the warm and cold seasons. Third, we conducted a sensitivity analyses among the healthiest group of women, excluding those with an asthma diagnosis or chronic bronchitis. Finally, we ran the models stratified by smoking status. We defined statistical significance as a *p*-value < 0.05. We conducted all analyses in SAS version 9.0 (SAS Institute Inc., Cary, NC, USA).

## Results

The sample of 272 women 18–42 years of age included 57% who self-identified as nonwhite (144 Hispanics, 2 blacks, 1 Asian, and 7 other race/ethnicity), 41% with less than high school education, and 18% who were current smokers (Table 1). Mean ± SD predicted annual BC exposure level was 0.62 ± 0.2μg/m^3^; mean FEV_1_ was 2.62 ± 0.4 L; mean FVC was 3.10 ± 0.4 L, and mean FEF_25–75%_ was 3.26 ± 0.8 L/sec. The IQR of BC was 0.22 μg/m^3^ (Table 1).

BC was associated with race/ethnicity and education in bivariate analyses and with smoking status. Hispanics and women with a high school education or less had higher BC levels than did their counterparts. Nonsmokers also had higher levels of predicted BC exposure than did current or former smokers. Lower levels of lung function were associated with a diagnosis of asthma or chronic bronchitis.

In adjusted analysis, BC (per IQR increase) was associated with a 1.1% decrease [95% confidence interval (CI), −2.5 to 0.3%] in FEV_1_, a 0.6% decrease (95% CI, −1.9 to 0.6%) in FVC, and a 3.0% decrease (95% CI, −5.8 to −0.2%) in FEF_25–75%_ (Table 2). Adjustment for smoking history, asthma, and chronic bronchitis slightly increased the effect estimates. Further adjustment for environmental tobacco smoke exposure, use of a gas stove for cooking, and occupational dust exposure did not change the effect estimates. In multilevel models adjusting for percent unemployed within census tract and living in a census tract where > 20% of households earned less than the federal poverty guidelines, the previously reported effect estimates actually increased slightly: FEV_1_, −1.44% (95% CI, −3.2 to 0.3%), FVC, −1.06% (95% CI, −2.7 to 0.5%), and FEF_25–75%_,−3.58% (95% CI, −7.2 to 0.06%).

**Figure 1:**
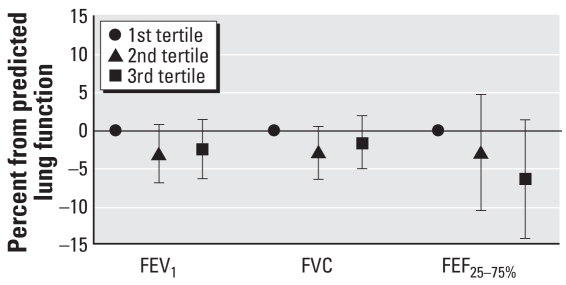
Linear regression models of predicted tertiles of BC by lung function measures (*n* = 272). All models were adjusted for age, race/ethnicity, education level, smoking history, asthma and chronic bronchitis, and year of assessment. Trend test: FEV_1_, *p* = 0.2; FVC, *p* = 0.3; FEF_25–75%_, *p* = 0.10.

To explore the potential for an exposure–response relationship, we conducted additional analyses using tertiles of BC exposure. In analyses adjusted for age, race/ethnicity, education level, smoking history, asthma and chronic bronchitis, and year of assessment (Figure 1), the highest BC exposure group (mean, 0.8 μg/m^3^) had decreases in FEV_1_, FVC, and FEF_25–75%_ compared with the lowest tertile group (mean, 0.5 μg/m^3^), although these differences were not statistically significant (*p* > 0.05). Again, further adjustment for all other covariates did not change these findings significantly.

As sensitivity analyses, we conducted analyses stratified by length of residence (data not shown). Among women who had lived in their residence for > 2 years, effect estimates did not change from those previously reported for the entire group. We also conducted analyses stratified by season. The effect estimates and 95% CIs for a one-IQR-range increase in BC during the summer months are, for FEV_1_, −1.32% (95% CI, −2.8 to 0.2%); FVC, −0.70% (95% CI, −2.1 to 0.2%); and FEF_25–75%_, −3.34% (95% CI, −6.3 to −0.4%); and for the winter are, for FEV_1_, −0.83% (95% CI, −2.2 to 0.5%); FVC, −0.52% (95% CI, −1.7 to 0.7%); and FEF_25–75%_, −2.73% (95% CI, −5.4 to −0.1%).

In regression analyses on the healthiest group of women, those without an asthma diagnosis or chronic bronchitis, the effect estimates of BC on lung function were very similar to the effect estimates found in the full cohort. The group with the highest BC exposure had decreases in FEV_1_ (−2.6%; 95% CI, −6.6 to 1.4%), FVC (−1.7%; 95% CI, −5.4 to 2.1%), and FEF_25–75%_ (−6.9%; 95% CI, −15.0 to 1.2%) compared with the lowest tertile group.

We explored the potential of smoking history to act as a modifier of the association of interest. We noted significant interactions (*p* < 0.05) between BC and current smoking status and former smoking status for both FEV_1_ and FEF_25–75%_, so we repeated our adjusted linear regression models stratifying by smoking history (Table 3). We noted no significant effects of BC exposure among current smokers, but significant reductions in FEV_1_, FVC, and FEF_25–75%_ among former smokers (−4.4%, −3.1%, and −8.9%, respectively; all *p* < 0.05). Among never smokers, BC predicted a significant decrease in FEF_25–75%_ (4.4% decrease, *p* < 0.05) and decreases in FEV_1_ and FVC, but these did not reach statistical significance.

## Discussion

In this urban community–based cohort study, we found that predicted long-term BC exposure was associated with decreased lung function among younger women, after adjusting for smoking history, sociodemographics, and past medical history. We noted decreases across all three measures of lung function even after excluding women with a diagnosis of asthma or chronic bronchitis. In the overall sample, the most robust finding was a significant association between increased BC exposure and reductions in FEF_25–75%_, which is an indicator of small-airway disease leading to airway obstruction. This corroborates recent findings from Churg et al. (2003), who examined lung biopsy specimens under light microscopy comparing those from nonsmoking adult subjects with high and low air pollution exposure, documenting that long-term exposure to ambient PM was associated with small airway remodeling. Notably, the observed effects on FEF_25–75%_ were even stronger among nonsmokers in the stratified analysis. We also noted stronger effects in summer compared with winter months. Because windows are more likely to be closed and ventilation lower in the winter than in the summer, we think that the observed difference can most likely be explained by exposure differences.

Our results are comparable with other research relating traffic exposures to lung function among women. Recently, the Atherosclerosis Risk in Communities study, a community-based cohort, showed that increased traffic density and distance to major roadways were associated with reduced FVC and FEV_1_ among women (Kan et al. 2007). In other studies also focusing in women, increased exposure to PM ≤ 10 μm in aerodynamic diameter was associated with reduced FEV_1_ and FVC and increased risk of chronic obstructive pulmonary disease (COPD) (Schikowski et al. 2005). Sekine et al. (2004) reported decreased lung function among women living in Tokyo, Japan, who were exposed to automobile exhaust compared with women living in other cities with lower traffic exposures. To estimate traffic exposure, studies generally have relied on measures of traffic density and residence distance from roadway (Kan et al. 2007; Schikowski et al. 2005; Sekine et al. 2004), whereas other studies estimate PM exposure using area-level monitors (Schikowski et al. 2005). Our study goes beyond this by using estimated exposure to traffic particles from a validated model. Because these exposure estimates are both specific for traffic particles and based on monitoring data, this gives greater assurance that the association we observed is not entirely attributable to some other attribute of the GIS traffic estimates.

In stratified analyses, we noted stronger effects among former smokers than among current smokers. Other studies have suggested an interaction between smoking and air pollution exposure (Nyberg et al. 2000). We found no significant associations among current smokers. A “healthy smoker” effect has previously been noted for traffic-related air pollution (Nyberg et al. 2000). Although former smokers may have quit smoking because of respiratory symptoms and/or health concerns, current smokers may be less sensitive to the effects of tobacco smoke and, subsequently, to air pollution. Furthermore, physiologic changes among smokers (i.e., thickening of bronchial mucosa) may make them less susceptible to additional pollutants (O’Neill et al. 2003). The lack of an effect on lung function among current smokers could also be attributed to factors related to race and SES; for example, of the current smokers in our study, 93% self-identified as white and had lower BC exposures than did their Hispanic/black counterparts. Lower SES can contribute to exposure differentials in traffic-related pollution as well as influence other factors (i.e., stress, nutrition, access to health care), which can enhance susceptibility to air pollution (O’Neill et al. 2003; Romieu 2005; Wright et al. 2005).

As in any epidemiologic study, we acknowledge a number of limitations. As is typical with longitudinal studies, there was significant reduction in the sample available from the original cohort over time. The nonparticipation of subjects from the longitudinal study may be seen as a limitation, although we noted no differences based on race/ethnicity, age, education, or smoking status between those who had lung function assessed and those who did not. Thus, this likely did not influence our findings. Although we are able to adjust for a number of factors associated with lung function and air pollution, it is still possible that the associations found in this study can be attributable to unmeasured or residual confounding, perhaps most notably due to SES. In addition to adjusting for mother’s education level, the present study is somewhat restricted regarding SES given that all families were recruited from one neighborhood health center in Boston, restricting the variability of individual-level SES in this population. This limited variability reduces the potential for confounding. Moreover, when we further adjusted for neighborhood-level indicators of SES, the observed effects were actually strengthened.

Although we attempted to capture BC exposure from all residential addresses, we may be missing exposures at work and/or other locations where the participants spend a portion of their time. However, this potential misclassification of exposure is nondifferential with respect to the outcome, so it is unlikely to account for the associations found in this study. Furthermore, exposure studies using personal monitors indicate that home exposures are the most important in predicting personal exposure (Liu et al. 2003; Rohas-Bracho et al. 2000). Other studies (Sarnat et al. 2002) have shown that home indoor concentrations of PM of outdoor origin are highly correlated with outdoor concentrations. In another study (Brown 2006), the personal exposures of the working spouses of persons with chronic illnesses have been shown to be highly correlated with their spouse’s personal exposures. Taken together, we believe these studies indicate that personal exposures to ambient particles are driven primarily by exposures at home. Moreover, we attempted to capture BC exposure from all residential addresses when participants moved.

Another limitation of this study is the use of predicted exposure, rather than observed measurements taken outside the residences of the study participants. Because the latter is not practical in a large community-based study, we decided to use all available exposure data and advanced modeling approaches to predict the missing exposure at the residences of the participants. This approach has become very popular in recent years. A potential statistical issue that arises when using spatiotemporal predictions of exposure is that these quantities are uncertain rather than measured quantities, which could bias the resulting health effect estimates. In a previous study (Gryparis et al. 2006), we found that the use of predictions from spatial exposure models induces a Berkson-type measurement error. It is well known that in the Berkson-type measurement error framework, the use of the error-prone covariate results in unbiased regression parameter estimates, but the associated standard errors are no longer valid. Gryparis et al. (2006) showed that the use of predictions from spatial exposure models results in unbiased parameter estimates for the association between the predicted exposure and the observed health outcome. The issue with this approach is that the standard errors for the parameter of interest might be incorrect. In such a case, we would expect larger standard errors for the parameter of interest. Last, we have no information on other spatially variable pollutants (i.e., nitrogen dioxide, ozone) to compare with our results.

In summary, we found evidence of an association between BC, a marker of traffic pollution, and lung function among women. Risk factors contributing to lung function are of interest given that lung function is important to the development of COPD in later life (Spurzem and Rennard 2005). The public health implications are substantial, because COPD is projected to be the fourth leading cause of death worldwide by the year 2020 (Mannino 2005).

## Figures and Tables

**Table 1:** Sample demographics, environmental exposures, and lung function measures (*n* = 272).

Measure	Value
Demographics	
Age [years (mean ± SD)]	26.9 ± 5.3
Race/ethnicity [no. (%)]	
Hispanic/black/other^a^	154 (56.6)
White	118 (43.4)
Education level [no. (%)]	
Some college/technical school	60 (22.1)
High school graduate	100 (36.8)
Less than high school	112 (41.2)
Exposure and medical history [no. (%)]	
Tobacco exposure	
Never smoker	163 (59.9)
Former smoker	61 (22.4)
Current smoker	48 (17.7)
Environmental tobacco exposure	150 (55.2)
Occupational dust exposure	68 (25.0)
Gas stove used for cooking	212 (77.9)
Length of residence > 2 years	112 (41.2)
Asthma or chronic bronchitis	23 (8.5)
Lung function and predicted BC (mean ± SD)	
FEV_1_ (L)	2.62 ± 0.39
FVC (L)	3.10 ± 0.44
FEF_25–75%_ (L/sec)	3.26 ± 0.82
BC (μg/m^3^) average	0.62 ± 0.15

a Includes 144 Hispanics, 2 blacks, 1 Asian, and 7 women who self-identified as other race/ethnicity.

**Table 2:** Linear regression models of predicted BC (average summer and winter) by lung function measures: effect estimate [% (95% CI)] for change in percent predicted lung function per IQR increase in BC (0.22 μg/m^3^) (*n* = 272).

BC model	FEV_1_	FVC	FEF_25–75%_
Adjust demographics^a^	−1.08 (−2.5 to 0.3)	−0.62 (−1.9 to 0.6)	−2.97 (−5.8 to −0.2)
Adjust above + tobacco^b^	−1.10 (−2.5 to 0.3)	−0.62 (−1.9 to 0.6)	−3.04 (−5.9 to −0.2)
Adjust above + asthma + bronchitis	−1.09 (−2.5 to 0.3)	−0.62 (−1.9 to 0.6)	−3.03 (−5.8 to −0.3)

a We adjusted all models for age, race/ethnicity, education level, and year of assessment.

b Tobacco = current and past smoking.

**Table 3:** Linear regression models of predicted BC (average summer and winter) by lung function measures, stratified by smoking status (*n* = 272): effect estimate [% (95% CI)] for change in percent predicted lung function per IQR increase in BC (0.22 μg/m^3^).

BC model	FEV_1_	FVC	FEF_25–75%_
Current smokers (*n* = 48)	0.62 (−2.1 to 3.4)	0.64 (−2.0 to 3.3)	2.63 (−3.7 to 8.9)
Former smokers (*n* = 61)	−4.40 (−7.8 to −1.0)	−3.11 (−6.1 to −0.2)	−8.78 (−14.7 to −2.9)
Nonsmokers (*n* = 163)	−0.98 (−2.9 to 0.9)	−0.32 (−2.0 to 1.4)	−4.39 (−8.1 to −0.6)

a We adjusted all models for age, race/ethnicity, education level, asthma/chronic bronchitis diagnosis, and year of assessment.
